# Supplementary material for: Controlled manipulation of oxygen vacancies using nanoscale flexoelectricity
Source: Nat Commun. 2017 Sep 20;8:615. doi: 10.1038/s41467-017-00710-5 (PMC5607007; doi:10.1038/s41467-017-00710-5)
Supplement: Supplementary file 1 — Supplementary Information [file 41467_2017_710_MOESM1_ESM.pdf]

Description of Supplementary Files

File Name: Supplementary Information
Description: Supplementary Figures, Supplementary Table, Supplementary Notes, Supplementary Discussion and Supplementary References

File Name: Peer Review File
Description:

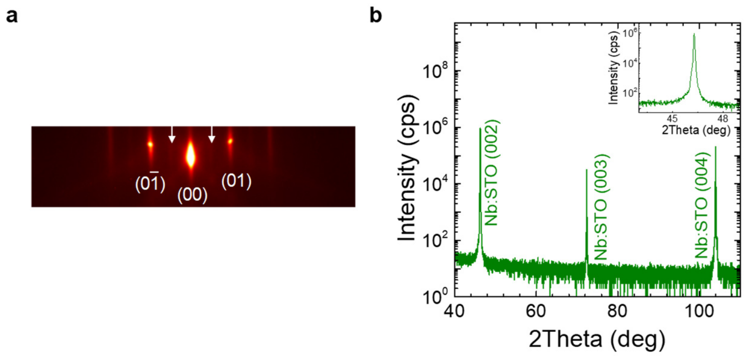

Supplementary Figure 1 | Structural characterisation of STO film. a, Reflection high energy electron diffraction (RHEED) pattern along the [100]-azimuth. This image was obtained after the growth of the 120-uc-thick $SrTiO_3$ (STO) film on the Nb:STO substrate. The arrows mark the half-order Bragg reflexes, which typically indicate of a (2x1)-type surface reconstruction. **b,** Symmetric XRD spectrum of the film. The film and substrate peaks are indistinguishable up to the 4th-order Bragg Reflex. This confirms stoichiometric film growth. The inset magnifies the XRD spectrum around the (002) peak.

Supplementary Note 1: Characterisation of $\mathrm{V_O^{\cdot\cdot}}$ diffusion using the KPFM technique

Here, adopting a step-by-step approach, we will elaborate on the application of Kelvin probe force microscopy (KPFM) to study the diffusion of oxygen vacancies ($\mathrm{V_O^{\cdot\cdot}}$) and determine the corresponding coefficient of diffusion (D). First, using the 14-uc-thick $SrTiO_3$ (STO) film as a model system, we discuss how to calculate the degree of equilibrium, $S(t)$, from KPFM images. As we discussed in the main text, the first step of this study was enriching a selected area of the STO surface with $\mathrm{V_O^{\cdot\cdot}}$ by poling with a tip bias of -5 V. Then, we performed a series of KPFM measurements over a span of six hours, wherein KPFM images were acquired at regular intervals of approximately one hour. Supplementary Figs. 2a-c show three KPFM images from this series. As time elapsed, the darker, $\mathrm{V_O^{\cdot\cdot}}$-enriched region exhibits a change in contrast, which indicates a time-dependent change in the vacancy concentration.

The time evolution of vacancy concentration can be quantified using the degree of equilibrium. This dimensionless quantity can be defined as follows:

$$S(t) = \frac{[V_b(t) - V_{min}(t)] - [V_b(t_0) - V_{min}(t_0)]}{[V_b(t_\infty) - V_{min}(t_\infty)] - [V_b(t_0) - V_{min}(t_0)]} \qquad (1)$$

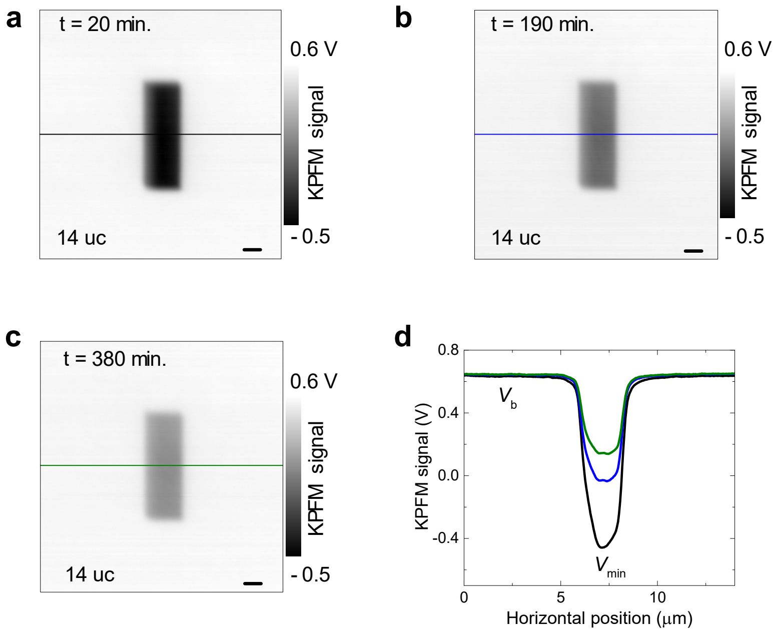

Supplementary Figure 2 | Time evolution of KPFM signal. a-c, KPFM images around a $V_O^{\cdot\cdot}$-enriched surface region of a 14-uc-thick STO film at 20 minutes (**a**), 190 minutes (**b**), and 380 minutes (**c**) after the $V_O^{\cdot\cdot}$-enrichment. The $V_O^{\cdot\cdot}$ -enrichment was performed by poling the pristine surface with a tip bias of -5 V. **d,** Line profiles from KPFM images in (**a**)-(**c**). Positions used to extract the baseline (V_b) and minimum (V_{min}) KPFM signal for calculating the equilibrium degree, $S(t)$, are indicated on the graph. The scale bar in **a-c** represents 1 μm.

where $V_b(t)$ and $V_{min}(t)$ denote the baseline and minimum KPFM signal, respectively. Supplementary Fig. 2d plots horizontal line profiles across the KPFM images in Supplementary Figs. 2a-c. These profiles consist of a plateau and central minimum, which we used to extract $V_b(t)$ and $V_{min}(t)$, respectively. At any given time, t, $[V_b(t)-V_{min}(t)]$ corresponds to the concentration of $V_O^{\cdot\cdot}$ within the $V_O^{\cdot\cdot}$ -enriched region. In Supplementary Equation (1), t_0 refers to the time lag between the $V_O^{\cdot\cdot}$-enrichment process and the time the first KPFM image was acquired. Meanwhile, t_∞ refers to the time required for the vacancy concertation within the enriched region to equilibrate with the pristine concentration: $[V_b(t_\infty)-V_{min}(t_\infty)] = 0$. Following this approach and by analysing all KPFM images from the series, we calculated $S(t)$ for the 14-uc- and 120-uc-thick STO films, which are shown by symbols in Fig. 1e of the main text. From the definition in Supplementary Equation (1), it is clear that $S(t)$ essentially describes how the surface approaches the equilibrium state after the electrical poling. As we outlined in the main text, the surface equilibrates through diffusion of $V_O^{\cdot\cdot}$.

The $V_O^{\cdot\cdot}$ diffuse both along and perpendicular to the film surface. First, to demonstrate the diffusion along the film surface, we show in Supplementary Figs. 3a-b the KPFM images of the 120-uc thick STO film from Fig. 1d of the main text. Supplementary Figs. 3c and e show KPFM profiles along the horizontal and vertical linecuts in Supplementary Figs. 3a-b. These profiles exhibit a broadening that is marked by arrows. For quantifying this broadening, in Supplementary Figs. 3d and f we plot the 1st derivative of these profiles. From a Gaussian fitting (not shown) we found that the full width at half maxima (FWHM) of these derivative profiles increases, in either direction, from about 0.5 to 0.8 μm.

The FWHM of the derivative profile is sensitive to the sharpness of boundaries between the $V_O^{\cdot\cdot}$-enriched and pristine regions, and the spatial resolution of the KPFM technique. Since the KPFM images in Supplementary Figs. 3a-b were obtained using identical scanning parameters, and with the same scanning probe microscope (SPM) tip, the spatial resolutions of these two measurements should be identical. Hence, the increase of the FWHM suggests the sharpness of boundaries decreases with time–the $V_O^{\cdot\cdot}$-enriched region laterally expands. This lateral expansion can be attributed to the in-plane diffusion of vacancies.

The KPFM profiles of the 14-uc thick STO film (Supplementary Fig. 2d), however, does not show a discernible broadening. The rapid relaxation of the KPFM contrast inhibits us detecting the broadening, even using the aforementioned FWHM-analysis.

Having elaborated that $V_O^{\cdot\cdot}$ diffuse along the STO surface that is clearly discernible in the thicker STO film; we would like to remark that a meaningful extraction of *D* from this in-plane diffusion is not possible. The poor spatial resolution of the KPFM technique does not allow us to quantify the lateral expansion of the $V_O^{\cdot\cdot}$-enriched region.

Notably, while calculating *S*(*t*) in Supplementary Equation (1), we defined the concentration of $V_O^{\cdot\cdot}$ at any given time, *t*, as $[V_b(t)-V_{min}(t)]$. We used the pristine region and the center of the $V_O^{\cdot\cdot}$-enriched region for extracting $V_b(t)$ and $V_{min}(t)$, respectively. Over time, a certain fraction of $V_O^{\cdot\cdot}$ those were initially located away from boundaries, including those at the center, would certainly diffuse in the

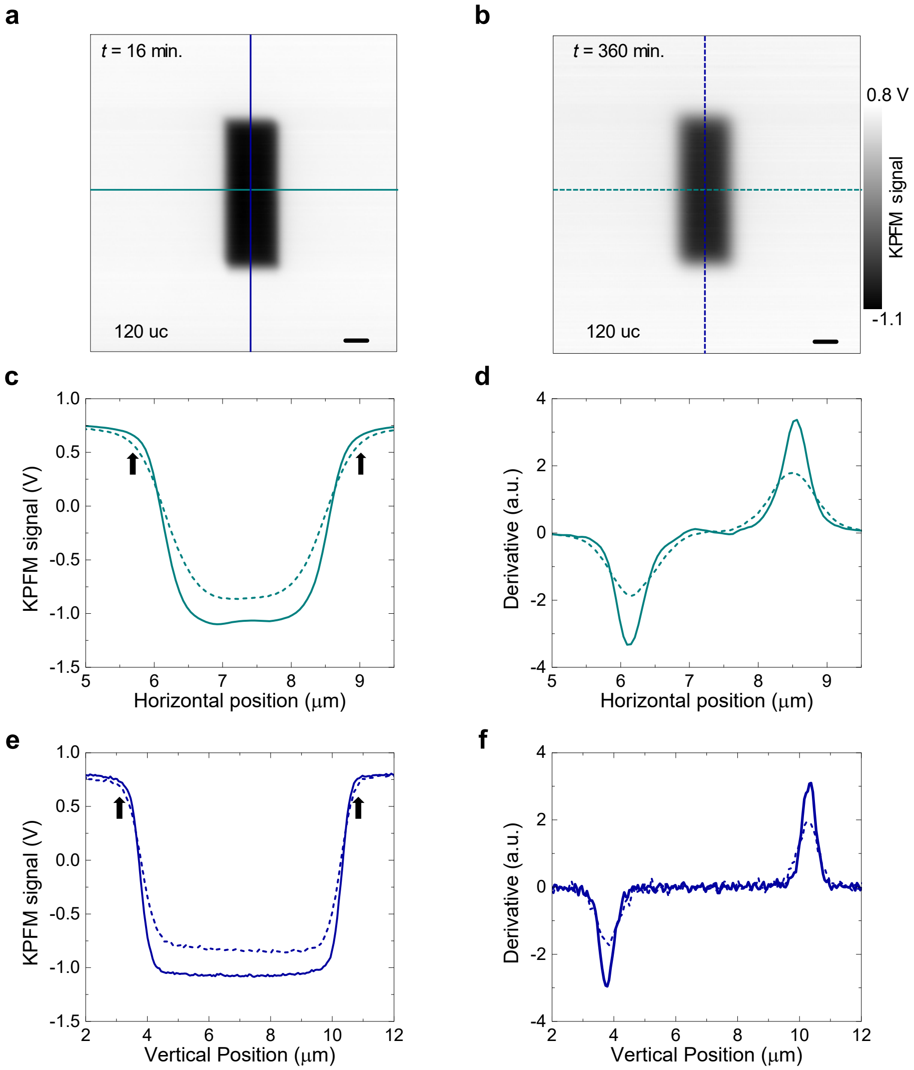

Supplementary Figure 3 | Evidence of in-plane diffusion of oxygen vacancies. a-b, KPFM images around a $V_{O}^{\cdot\cdot}$-enriched surface region of the 120-uc-thick STO films. The time lag between poling the pristine surface and the time of acquiring an image is indicated on the top right corner of KPFM images. Note that **a** and **b** are the same images as those in Fig. 1d of the main text. **c-d,** Horizontal potential profiles (**c**) and their first derivatives (**d**). **e-f,** Vertical potential profiles (**e**) and their first derivatives (**f**). These potential profiles are taken from the KPFM images in **a** and **b.** The arrows in figures (**c**) and (**e**) mark the broadening due to the in-plane diffusion of $V_{O}^{\cdot\cdot}$. The scale bar in **a-b** represents 1 μm.

lateral directions along the surface. On average this lateral diffusion, however, would not perturb the KPFM signal, and thus the $S(t)$. In contrast, owing to the high surface sensitivity of the KPFM technique, only the surface-bulk diffusion of $V_{O}^{\cdot\cdot}$ predominantly influences $S(t)$. In the following, we discuss how to extract the corresponding diffusion coefficient (D) from $S(t)$.

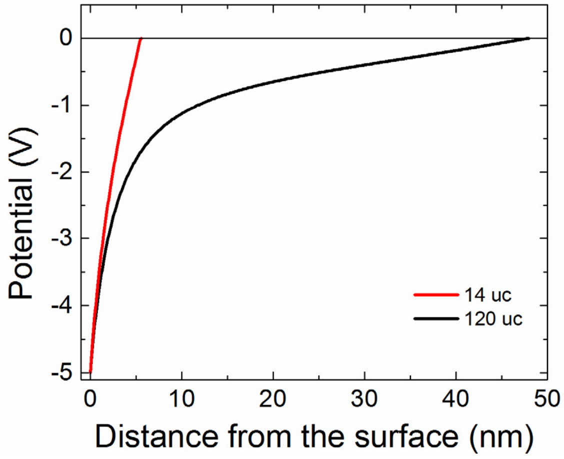

Supplementary Figure 4 | Simulated depth profiles of electrical potential. Simulated electrical potential profiles inside the 14-uc and 120-uc thick STO films under an applied tip bias of -5 V. This simulation was performed assuming that the top electrode is biased, while the bottom electrode is grounded, and the relative permittivity of STO is 10. The simulated potential profiles show a gradual decay of electric potential over the thickness.

First, we note that the electrical poling was performed with a tip bias of -5 V, while the bottom electrode (Nb:STO) was grounded. In Supplementary Fig. 4, we compared the depth profiles of the simulated electric potential inside the STO films under the applied tip bias. In this simulation we assumed that the top electrode is biased: V (x = 0 nm) = -5 V and the bottom electrode is grounded: V (x = film thickness in nm) = 0 V. Supplementary Fig. 4 shows that the electrical potential gradually decays over the film thickness. The applied tip bias would perturb the equilibrium distribution of $V_O^{\cdot\cdot}$ in STO. Based on the simulated potential profiles shown in Supplementary Fig. 4, we can therefore argue that this perturbation spans the entire film thickness. In this non-equilibrium state, vacancies in larger concentration would accumulate around the surface region. Upon removing the applied bias, the system would relax to its equilibrium state through the vacancy diffusion.

Second, we note a salient feature of $V_O^{\cdot\cdot}$ diffusion, which is associated with the electrically charged nature of $V_O^{\cdot\cdot}$. Molecular dynamics simulations of $V_O^{\cdot\cdot}$-diffusion in STO suggest that during diffusion, the repulsive interaction between charged $V_O^{\cdot\cdot}$ inhibits them from moving independently[1].

Summing up above considerations, we can argue that the tip bias applied during the poling perturbs the equilibrium $V_O^{\cdot\cdot}$-distribution of the whole STO thickness under the poled area. This

perturbed volume equilibrates through diffusion of vacancies, which occurs both along the out-of-plane and in-plane directions. The KPFM technique being surface sensitive, the surface-bulk diffusion of $V_O^{\cdot\cdot}$ predominantly causes the KPFM signal or equivalently *S(t)* to change over time. However, owing to the correlated movement of $V_O^{\cdot\cdot}$ during the out-of-plane diffusion of $V_O^{\cdot\cdot}$, the time evolution of the KPFM signal or *S(t)* effectively describes how the perturbed volume under the poled area relaxes to the equilibrium state.

Following the rationale above, we calculated the diffusion coefficient (*D*) by fitting the time evolution of *S*(*t*) with Fick's 2nd law of diffusion, which is approximated for small time and one-dimensional cases as follows: $S(t) = \frac{4\sqrt{(t-t_0)D}}{L\sqrt{\pi}}$. Here, *L* refers to the film thickness, which equilibrates over time through diffusion[2–4]. Note that the use of this one-dimensional approximation is justified because only the out-of-plane diffusion of vacancies influences *S*(*t*). Another point worth mentioning concerns the thickness (*L*) dependence of *S(t)*, which for a given *D* value determines how fast the film equilibrates. The inverse proportionality between *L* and *S*(*t*) justifies the faster temporal evolution of *S*(*t*) for the 14-uc thick STO than for the 120-uc thick STO (Fig. 1e in the main text), albeit the calculated *D* values for these two films are very similar.

Supplementary Note 2: The effect of mechanical scan on the background in the pristine region.

To quantify the lateral motion of $V_O^{\cdot\cdot}$ along with the tip, in Figs. 2e and 4f of the main text, we used a nonzero normalised vacancy concentration (NVC) in pristine regions along the lines M_L, M1, and M4 as our background. Furthermore, we argued that a mechanical scan does not alter this background. In order to support this argument, we performed mechanical scans around two boundaries between a $V_O^{\cdot\cdot}$-enriched region and a pristine region of STO surface, as indicated in Supplementary Fig. 5a by boxes A and B. These scans were performed with a sharp tip (*R* = 25 nm) under a contact force of 5 μN. While box A was scanned, the fast-scan axis was normal to the boundary between the $V_O^{\cdot\cdot}$-enriched region and the pristine region. Therefore, the tip crossed this

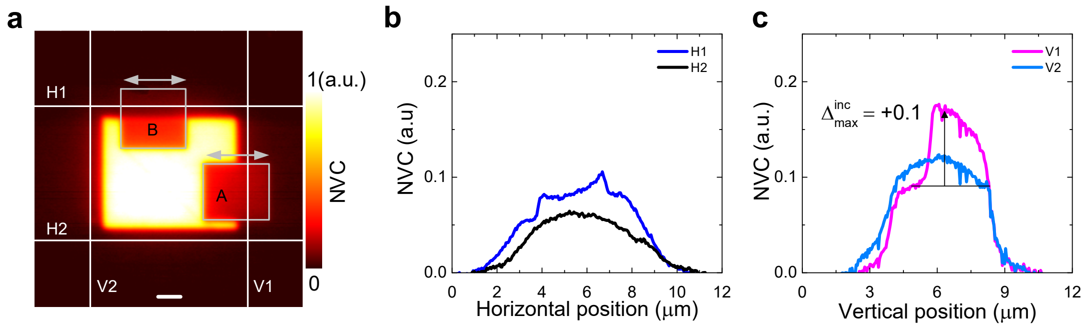

Supplementary Figure 5 | The scan direction-dependent lateral motion of vacancies. a, The normalised vacancy concentration (NVC) map after mechanical scans were performed using a sharp SPM tip with a contact force of 5 μN. Scans were performed within areas marked by boxes A and B. Arrows mark the corresponding fast-scan direction. Prior to mechanical scans, the $V_O^{\cdot\cdot}$-enrichment was carried out by poling the pristine surface with a tip bias of -5 V. **b,** NVC profiles along the H1 and H2 lines in **a. c,** NVC profiles along the V1 and V2 lines in **a.** Δ^{inc}_{max} refers to the net gain in NVC along the line V1. H1, H2, V1 and V2 lines are placed at a distance 0.5 μm outside the $V_O^{\cdot\cdot}$-rich region, and NVC profiles are averaged over a 0.5 μm-wide averaging window. The scale bar in **a** represents 1 μm.

boundary while it moved back and forth. However, while box B was scanned, the fast-scan axis was parallel to the border. Thus, the tip first scanned the $V_O^{\cdot\cdot}$ -enriched region and then the pristine area. By comparing the resulting change in NVC within the pristine regions enclosed by boxes A and B, we would elaborate that the mechanical scan does not alter the background.

For this comparison, we profiled the NVC map along four sections: lines H1, H2, V1, and V2, as shown in Supplementary Fig. 5a. Supplementary Fig. 5b plots the horizontal NVC profiles H1 and H2, while Supplementary Fig. 5c shows the vertical NVC profiles V1 and V2. The NVC profiles H2 and V2 can be treated as reference profiles, which mark the intrinsic background outside the $V_O^{\cdot\cdot}$ -enriched part. First, we note that mechanical scans identically deplete the $V_O^{\cdot\cdot}$-enriched region within boxes A and B. By profiling (not shown), we estimated the resulting drop in NVC, Δ^{dec}_{max}> -0.5. However, a direct comparison between Supplementary Figs. 5b and c suggests that the change in NVC is negligible along H1. However, the increase in NVC is sizeable (Δ^{inc}_{max} = +0.1) along V1, as indicated by a vertical arrow in Supplementary Fig. 5c. These observations validate our argument that the mechanical scan does not alter the background within the pristine region of the NVC map.

The negligible effect of the mechanical scan on the NVC along line H1 (Supplementary Fig. 5b) highlights following points. First and foremost, the mechanical scan does not produce $V_o^{\cdot\cdot}$ on the STO surface. Second, the triboelectric charging or contact electrification of the surface due to friction during a mechanical scan is negligible. The presence of strong triboelectric charge would otherwise significantly alter the KPFM signal[5] and hence the NVC along H1. Taking into consideration these points, we can therefore argue that the enrichment of the pristine region by a mechanical scan is caused by $V_o^{\cdot\cdot}$ that laterally move with the tip from the $V_o^{\cdot\cdot}$-enriched region towards the pristine region.

Supplementary Note 3: PFM characterisation

In this section, using the piezoresponse force microscopy (PFM) characterisation, we show that our 120-uc-thick STO film is not ferroelectric. First, we note that pure STO is a quantum paraelectric material[6]. However, strain or chemical disorders can drive an STO film into the ferroelectric phase[7,8,9]. Based on our structural characterisation (Supplementary Fig. 1b), we can exclude these extrinsic contributions in our film. Supplementary Fig. 6 shows PFM images that were acquired after obtaining the KFPM image in Fig. 2a of the main text. Vertical (V) and lateral (L) PFM images were obtained using an AC excitation $V_{\text{peak-peak}}$ = 0.8 V and a contact resonance frequency in the range of 1.2-1.7 MHz. The uniform $V_o^{\cdot\cdot}$-enriched area exhibits a higher PFM amplitude and an 180˚ phase offset relative to the pristine region. On the other hand, changes in the PFM-amplitude and phase reversals are noticeable within the electrically and mechanically scanned parts of the $V_o^{\cdot\cdot}$-enriched region.

Remarkably, some features in these PFM images resemble that would be expected from a ferroelectric material, for example, the evolution of the PFM amplitude, particularly within the electrically scanned part. At first, the amplitude decreases, and then it reaches a minimum at applied bias V_{dc} = + 3 V and further increases as the bias increases. This can be clearly observed in Supplementary Fig. 6c, which shows the VPFM amplitude along line E of Supplementary Fig. 6a. Furthermore, the PFM phase also reverses at $V_{dc} \geq 3$ V. Similarly, a VPFM amplitude minimum and

an 180° phase reversal can be observed along line M (at contact force of 8.5 μN) in the mechanically scanned region. The LPFM images in Supplementary Figs. 6d, e, also contain similar features.

The co-existing amplitude minimum and phase-reversal in a PFM image are hallmarks of ferroelectric domain switching. This domain switching can occur when the electric (flexoelectric) field induced by the tip bias (contact force) exceeds the coercive field[10]. Therefore, one might infer that our STO film is ferroelectric. If this is true, then the ferroelectric polarisation (**P**) must be aligned along the [111] direction, which would then explain the coexisting VPFM and LPFM signals. However, such [111]-oriented **P** has neither been experimentally reported nor theoretically predicted in STO, neither in bulk nor in a thin film[11]. We therefore conclude that the origin of the PFM signal is non-ferroelectric.

To understand the physical origin of PFM signal, we note that for a given phase, Φ, the amplitude (A) of a PFM signal (= $A \cos\Phi$) can be expressed as follows:

$$A = A_{\mathrm{piezo}} + A_{\mathrm{el}} + A_{\mathrm{nl}}$$

$$= [d_{ij} + \mathrm{m}(V_{\mathrm{dc}} - V_{\mathrm{cs}}) + \mathrm{n}(V_{\mathrm{dc}} - V_{\mathrm{s}}^{\mathrm{av}})]V_{\mathrm{ac}} \quad (2)$$

In Supplementary Equation (2), the A_{piezo}, A_{el}, and A_{nl} terms refer to the piezoelectric, local and non-local electrostatic contributions, respectively[12]. For a given AC-excitation, the A_{piezo} term depends on the material-specific piezoelectric coefficient, d_{ij}. In contrast, the electrostatic contribution, A_{el} (A_{nl}) scales with the offset between the DC-bias, V_{dc}, applied to the probe assembly during a PFM scan and the contact surface potential V_{cs} (average surface potential observed by the cantilever, V_s^{av}). A non-zero surface potential can arise from surface charges formed during the poling with a biased tip or oxygen vacancy accumulation and the resulting change in the contact potential difference (CPD). Typically, a zero DC-bias (V_{dc} = 0 V) is used during PFM-imaging. Hence, a non-zero electrostatic contribution is always present in the PFM measurement. For ferroelectric materials with a large piezoelectric coefficient, $A_{\mathrm{piezo}} >> A_{\mathrm{el}}, A_{\mathrm{nl}}$, this electrostatic contribution can be neglected. In contrast, while imaging a non-ferroelectric and non-piezoelectric STO film with a zero piezoelectric coefficient (d_{ij}), the electrostatic contributions should dominate the PFM signal[13].

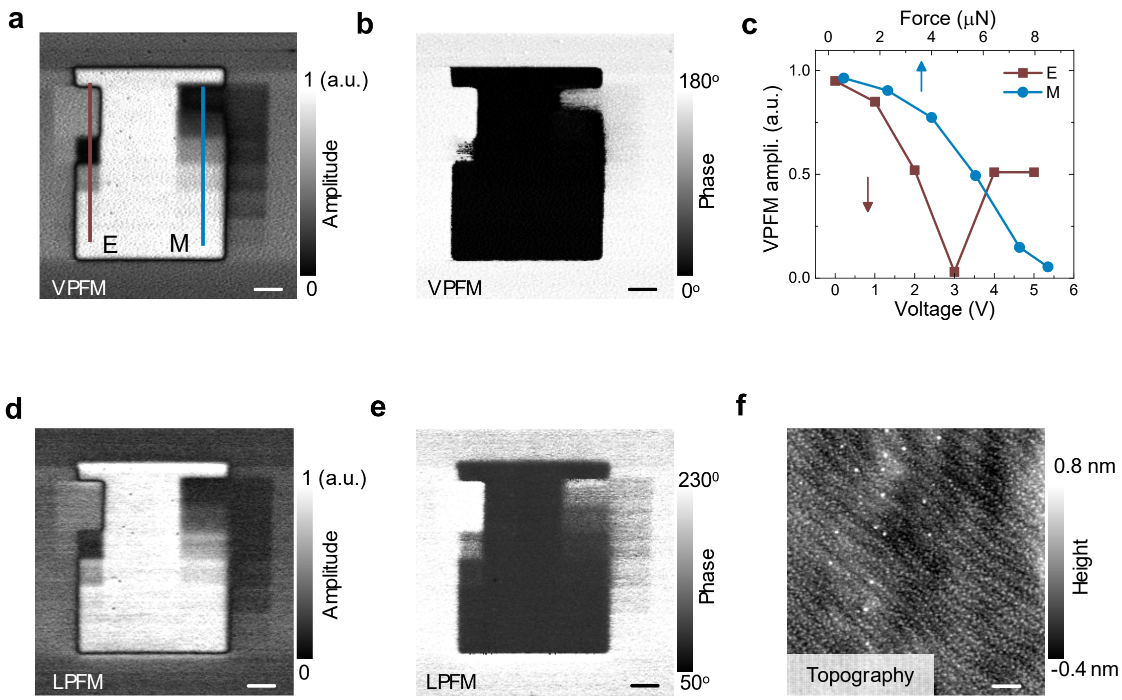

Supplementary Figure 6 | Piezoresponse force microscopy characterisation. a, The vertical PFM (VPFM) amplitude image. **b,** VPFM phase image. **c,** The variation of the VPFM amplitude along lines E and M in **a**. **d,** The lateral PFM (LPFM) amplitude image. **e,** LPFM phase image. **f,** Topography image that was acquired in the contact mode during the PFM measurement. These images were subsequently obtained after the KPFM image in Fig. 2a of the main text. No sign of surface deformation due to applied force is present in the topography image **f**. The scale bar in **a**,**b**,**d**,**e**, and **f** represents 1 μm.

Recently, it has been reported that STO films can simultaneously exhibit VPFM and LPFM responses in the presence of oxygen vacancies at the surface[2]. It that report, the PFM response was attributed to the electrostatic contribution due to a non-zero surface potential arising from accumulated vacancies. Our result is consistent with this report. Additionally, by considering the systematic decrease in NVC with applied bias and force from Fig. 2 of the main text, we can correlate the VPFM amplitude with the vacancy concentration. The bias $V_{dc} \geq 3$ V (contact force of 8.5 μN), for which the VPFM amplitude attains a minimum and the PFM phase reverses, corresponds to a drop in NVC ≥ 50%. All these observations therefore point towards a common electrostatic origin of the PFM signal and attest that our STO film is not ferroelectric.

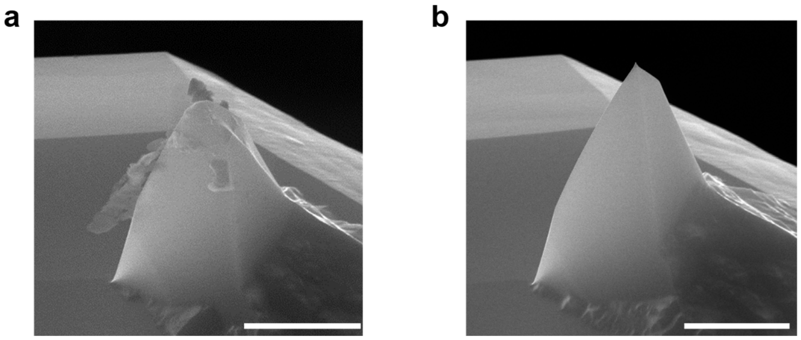

Supplementary Figure 7 | SEM images of SPM tips. a, Scanning electron microscope (SEM) image of the blunt tip that was used for the controlled manipulation of oxygen vacancies. We estimated the radius of curvature of this tip to be larger than 200 nm. **b,** The SEM image of an as-received SPM tip, with an estimated radius of curvature = 25 nm. The scale bar in **a**-**b** represents 5 μm.

Supplementary Note 4 : Theoretical modelling of vacancy redistribution under an applied force

To gain insight into the mechanical redistribution of oxygen vacancies, we numerically solved coupled time-dependent Ginzburg-Landau (TDGL) and Nernst-Planck equations. The time-dependent Ginzburg-Landau equation, which constitutes the basic building block of the phase-field simulation can be expressed as follows:

$$\frac{\partial \boldsymbol{P}}{\partial t} = -L\frac{\delta F}{\delta \boldsymbol{P}} \tag{3}$$

Here, $\boldsymbol{P}$ represents the polarisation vector, L is the kinetic coefficient, and F is the total free energy of the system. The energy functional F of the ferroelectric film can be expressed as follows:

$$F = \int(\alpha_{ij}P_iP_j + \alpha_{ijkl}P_iP_jP_kP_l + \frac{1}{2}c_{ijkl}\varepsilon_{ij}\varepsilon_{kl} - q_{ijkl}\varepsilon_{ij}P_kP_l - \frac{1}{2}E_i\left(\varepsilon_0\kappa_{ij}^{\mathrm{b}}E_j + P_i\right) + \frac{1}{2}g_{ijkl}\frac{\partial P_i}{\partial x_j}\frac{\partial P_k}{\partial x_l} + \frac{1}{2}f_{ijkl}(\frac{\partial P_k}{\partial x_l}\varepsilon_{ij} - \frac{\partial \varepsilon_{ij}}{\partial x_l}P_k))\,dV \tag{4}$$

Here, α_{ij} and α_{ijkl} denote the second- and fourth-order Landau coefficients, c_{ijkl} is the stiffness tensor, q_{ijkl} is the electrostrictive tensor, κ_{ij}^{b} is the background dielectric tensor, g_{ijkl} is the gradient energy coefficient, and f_{ijkl} is the flexocoupling coefficient in units of voltage (V). The numerical values of these coefficients are listed in Supplementary Table 1.

The flexoelectric contribution depends on the flexocoupling coefficients and force-induced strain-gradient (or stress-gradient). Additionally, there is a growing consensus that flexoelectricity could involve a strong contribution from the surface layer[14,15]. Although we did not actively control the surface termination during growth, it is likely that our STO film is TiO_2-terminated. We can indirectly support this argument from the RHEED pattern (Supplementary Fig. 1a), which consists of half-order Bragg reflexes that are similar to those obtained for a bare TiO_2-terminated Nb:STO substrate surface (images not shown). Furthermore, the atomic force microscopy (AFM) images (not included) of identically grown STO films, with thicknesses ranging from 14 to120-uc, consist of only single-unit cell (0.4 nm) steps. This implies that the STO surface is singly terminated. A mixed TiO_2 and SrO termination would otherwise result in half-unit cell steps. These observations led us to use flexocoupling coefficients that are theoretically derived using the first-principle calculation and assuming a TiO_2-terminated STO surface[14].

The contact force-induced stress-gradient is calculated by treating the sharp SPM tip as a rigid spherical indenter. The stress imparted by this indenter can be expressed as follows:

$$\sigma_{33}(r) = -\frac{3p}{2\pi a_{\mathrm{sph}}^2}\sqrt{1-\frac{r^2}{a_{\mathrm{sph}}^2}},\ r \le a_{\mathrm{sph}} \tag{5}$$

where p is the applied force, and a_{sph} is the contact radius. The contact radius a_{sph} is proportional to $p^{\frac{1}{3}}$, and r is the distance from the contact point.

For the blunt tip, a 12th order polynomial is used to construct a nearly flat contact surface underneath the tip[16]. The resulting additional surface displacement, δ, can be expressed as follows:

$$\delta(r) = \frac{br^{12}}{12},\ r < a_{\mathrm{flat}} \tag{6}$$

where b is a fitting constant, and a_{flat} is the contact radius of the blunt tip.

The second constituent equation in our model, namely, the Nernst-Planck equation provides insight into the response of $V_O^{\cdot\cdot}$ to flexoelectric polarisation. The Nernst-Planck equation has the following form:

$$\frac{\partial[V_O^{\cdot\cdot}]}{\partial t} = \nabla(D_{V_O^{\cdot\cdot}}\nabla[V_O^{\cdot\cdot}] + \mu_{V_O^{\cdot\cdot}}[V_O^{\cdot\cdot}]\nabla\phi) \tag{7}$$

where $D_{V_O^{\cdot\cdot}}$, $\mu_{V_O^{\cdot\cdot}}$, $[V_O^{\cdot\cdot}]$, and ϕ denote the diffusion coefficient, mobility, concentration of oxygen vacancies, and electrical potential, respectively. The diffusion coefficient and mobility are related through the Nernst-Einstein equation, i.e., $D_{V_O^{\cdot\cdot}} = \frac{k_B T}{z_{V_O^{\cdot\cdot}} q_0}\mu_{V_O^{\cdot\cdot}}$, where $z_{V_O^{\cdot\cdot}}$ and q_0 represent the valence of oxygen vacancy and elementary electron charge, respectively. The electrical potential ϕ is obtained by solving the Poisson equation, $-\nabla^2\phi = \rho_{\mathrm{total}}/\kappa_{\mathrm{r}}^{\mathrm{b}}\varepsilon_0$ under a short-circuit boundary condition at each time step. Here, the total charge density ρ_{total} consists of both the bound charge associated with the flexoelectric polarisation and the free charge, including electrons, holes and oxygen vacancies.

As a reference, we computed the concentration of electrons, holes and $V_O^{\cdot\cdot}$ from the work of R. Moos et al.[17]. Additionally, we assumed that the concentration of electrons and holes obey the Boltzmann statistics.

$$n = n_0 \exp\left(\frac{\phi q_0}{k_B T}\right) \tag{8}$$

$$p = p_0 \exp\left(\frac{-\phi q_0}{k_B T}\right) \tag{9}$$

In Supplementary Equations (8) and (9), n_0 and p_0 represent the reference concentration of electrons and holes, respectively. The reference values of the electron, hole, and oxygen vacancy concentration are listed in Supplementary Table 1. Note that all simulated NVC maps in this work are constructed by normalising the concentrations relative to this reference vacancy concentration.

The film surface and the substrate (Nb:STO) were assumed to block oxygen vacancies. Therefore, the Nernst-Planck equation (7) was solved by imposing the boundary condition that the flux ($J_{\mathrm{V_O^{\cdot\cdot}}}$) of oxygen vacancies at the film surface ($z = h_2$) and at the film-substrate interface ($z = h_1$) are both zero, i.e.,

$$J_{\mathrm{V_O^{\cdot\cdot}}}\Big|_{z=h_1,h_2} = -D_{\mathrm{V_O^{\cdot\cdot}}}\nabla[\mathrm{V_O^{\cdot\cdot}}] - \mu_{\mathrm{V_O^{\cdot\cdot}}}[\mathrm{V_O^{\cdot\cdot}}]\nabla\phi\Big|_{z=h_1,h_2} = 0 \tag{10}$$

Supplementary Table 1. Numerical values of parameters used in our theoretical modelling

Coefficients	Value	Units	Coefficients	Value	Units
α_{11}	1.83×10^{8}	C^{-2} m^{2} N	$D_{\mathrm{V_O^{\cdot\cdot}}}$	1.22868×10^{-15}	cm^{2} s^{-1}
α_{1111}	1.70×10^{9}	C^{-4} m^{6} N	$\mu_{\mathrm{V_O^{\cdot\cdot}}}$	9.56963×10^{-14}	cm^{2} V^{-1} s^{-1}
α_{1122}	1.37×10^{9}	C^{-4} m^{6} N	n_0	9.12187×10^{14}	cm^{-3}
c_{11}	3.36×10^{11}	N m^{-2}	p_0	1.79036×10^{14}	cm^{-3}
c_{12}	1.07×10^{11}	N m^{-2}	$[\mathrm{V_O^{\cdot\cdot}}]$	3.66576×10^{14}	cm^{-3}
c_{44}	1.27×10^{11}	N m^{-2}	p	4	μN
q_{11}	0.04581	C^{-2} m^{4}	a_{sph}	8	nm
q_{12}	-0.0135	C^{-2} m^{4}	a_{flat}	15	nm
q_{44}	0.0096	C^{-2} m^{4}	V_{bias}	1	V
g_{11}	1.0×10^{-11}	J m^{3} C^{-2}	f_{11}	0.8	V
g_{12}	0.5×10^{-11}	J m^{3} C^{-2}	f_{12}	-2.63	V
g_{44}	0.5×10^{-11}	J m^{3} C^{-2}	f_{44}	-2.01	V
$\kappa_{\mathrm{r}}^{\mathrm{b}}$	10	1			

Supplementary Note 5 : Simulation of the redistribution of oxygen vacancies under a positive tip bias

Our experiment indicates that $V_O^{\cdot\cdot}$ migrate from the surface to bulk in response to a positive bias from the SPM tip. However, in contrast to the mechanical case, $V_O^{\cdot\cdot}$ do not move laterally with the tip (Fig. 2 in the main text). To understand this behaviour of vacancies, we performed phase-field simulations under a positive tip bias. The surface of STO was assumed to be traction-free, and the electrical potential, ϕ, at the surface was approximated by the following Lorentzian distribution:

$$\phi_{\mathrm{surf}}(r) = \phi_0(\frac{\gamma^2}{\gamma^2+r^2}) \tag{11}$$

Here, ϕ_0 represents the applied bias voltage on the tip, γ is the half-width at half-maximum of the applied bias, and r is the distance from the contact point. Only the bound charge associated with the electric field-induced polarisation was considered when solving the Poisson equation to obtain the electrical potential distribution. An identical boundary condition, as given by Supplementary Equation (10), was used to solve the Nernst-Planck equation.

With an applied bias $\phi_0 = 1$ V and $\gamma = 10$ nm, the computed distributions of out-of-plane (E_z^{bias}) and in-plane (E_x^{bias}) components of the electric field on the film surface are plotted in Supplementary Figs. 8a and b, respectively. The resulting redistribution of vacancies can be visualised from the NVC map in Supplementary Fig. 8c. Because the E_z^{bias} component acting underneath the tip is directed downward, it depletes $V_O^{\cdot\cdot}$ from the contact junction within a circular region with a radius of approximately 20 nm. In contrast, though the in-plane electrical field may also drive the oxygen vacancies toward lateral directions, our simulation does not show any accumulation of $V_O^{\cdot\cdot}$ on the surface. This can be understood by noting that E_x^{bias} field is an order of magnitude smaller than E_z^{bias}. Additionally, unlike the force-induced case shown in Fig. 3 of the main text, there is no inwardly (upwardly) directed in-plane (out-of-plane) electric field component around the contact edge that could trap vacancies on the surface. Our simulation therefore qualitatively accounts for the observed out-of-plane migration and the absence of in-plane motion of oxygen vacancies under an applied positive bias.

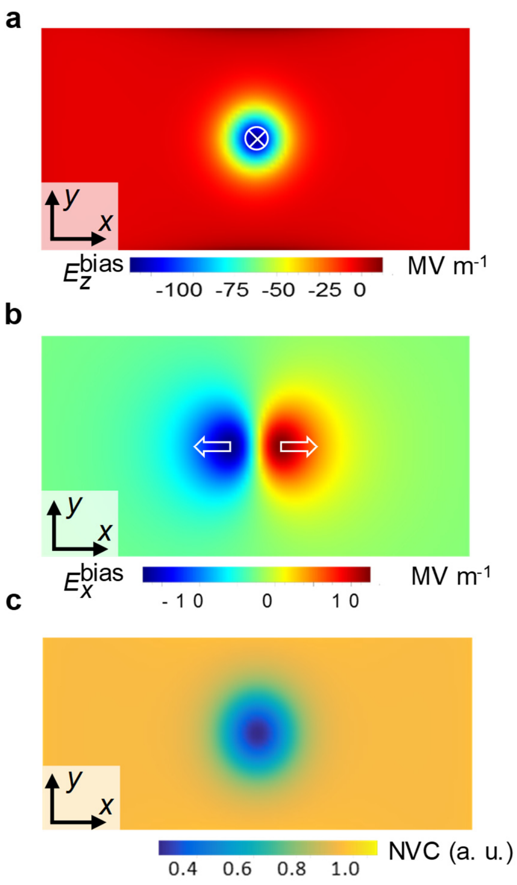

Supplementary Figure 8 | Simulated electric field and vacancy redistribution under a positive tip bias. a-b, Simulated electric field under a tip bias of +1 V. The in-plane distribution of the z-component, E_z^{bias} (**a**) and the x-component, E_x^{bias} (**b**). The y-component of the electric field has a similar distribution to E_x^{bias} but is rotated by 90° in the x-y plane. **c,** Simulated NVC map under this electric field. The NVC map shows a strong depletion underneath the tip and no lateral accumulation on the surface.

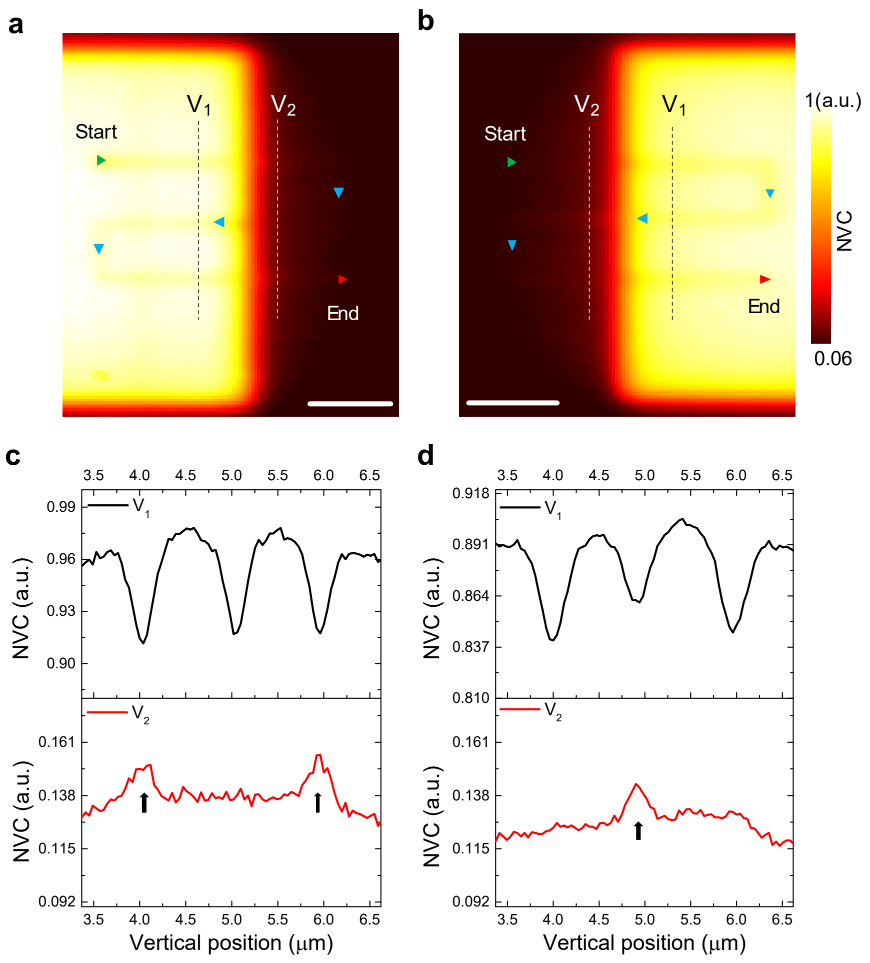

Supplementary Figure 9 | Oxygen vacancy redistribution by a single line scan. a-b, The normalised vacancy concentration (NVC) maps after mechanical scans were performed with a load of 6 μN across the left (**a**) right (**b**) boundaries between a vacancy enriched and pristine regions. We used the 120-uc thick STO film and a sharp tip for this experiment. During these scans, we traced the tip following a predefined defined path, which is marked by small triangles. The start (end) of the trace is marked by the green (red) triangle. **c-d,** NVC profiles along lines V_1 and V_2 in the NVC map in **a** (**c**) and **b** (**d**). The NVC profile along the line V_1 (V_2) shows a decrease (increase) of $V_O^{\cdot\cdot}$ concentration. The vertical arrows in the lower panel of **c** and **d** mark the increase in the vacancy concentration in the pristine regions. This study demonstrates the feasibility of laterally moving vacancies with a single trace. Furthermore, this study elaborates that irrespective of the scan direction, the tip laterally moves $V_O^{\cdot\cdot}$ from the $V_O^{\cdot\cdot}$-enriched to the pristine region and depletes the former region. Note that the high background inhibits us detecting the lateral motion of vacancies within the $V_O^{\cdot\cdot}$-enriched region. Another notable feature concerns the tip's trace from the pristine towards $V_O^{\cdot\cdot}$-enriched region, which does not enrich the pristine region. This implies that the mechanical scan does not create $V_O^{\cdot\cdot}$ on the STO surface, and also rules out any triboelectric charging of the surface. These arguments are in line with those we discussed in Supplementary Note 2. The scale bar in **a** represents 1 μm.

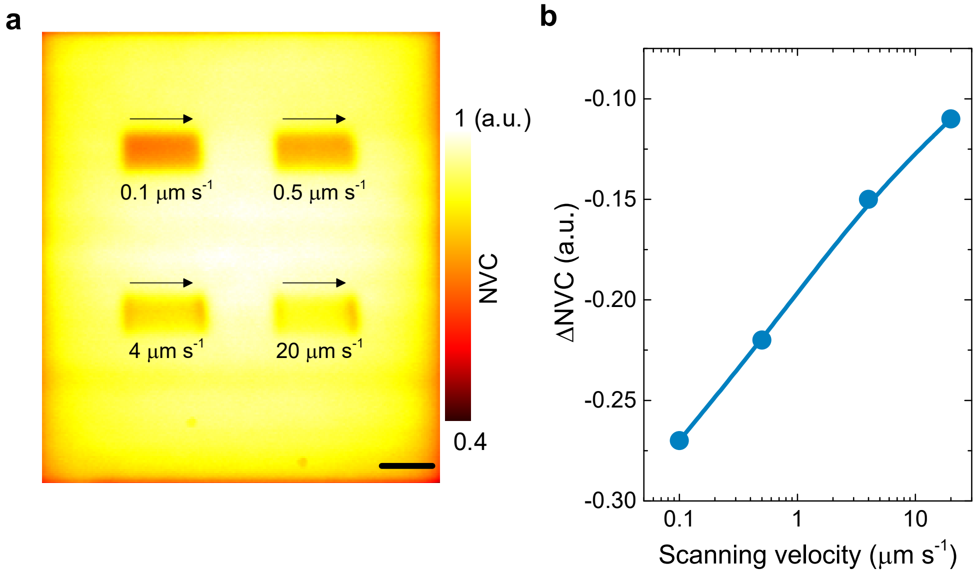

Supplementary Figure 10 | The scanning velocity dependence of vacancy depletion. **a**, The normalised vacancy concentration (NVC) map after mechanical scans were performed with different scanning velocities (0.1-20 μm s^{-1}) within a $V_O^{\cdot\cdot}$-enriched region. We used the 120-uc thick STO film and a sharp tip for this experiment. Scans were performed over a 1x0.5 μm^2 area with a contact force of 6 μN. The force was applied only during the trace, and the tip was lift-off during the retrace. Fives lines were traced from left-right as marked by horizontal arrows. **b**, Plot of the background subtracted NVC (ΔNVC) as a function of scanning velocity. The solid line is a guide for eyes. Notably, the high background within the $V_O^{\cdot\cdot}$-enriched region inhibits us detecting the lateral motion of $V_O^{\cdot\cdot}$ with the tip. Thus, this experiment only allows us characterising the surface-bulk migration $V_O^{\cdot\cdot}$ as a function of scanning velocity. Figure **b** shows that the net drop in vacancy concentration monotonically increases with decreasing scanning velocity. This implies the longer the tip stays in contact with the $V_O^{\cdot\cdot}$-enriched surface, the larger number of vacancies migrate into the bulk. Note an increased depletion of vacancies around the left and right sides, which is particularly discernible for rectangles scanned with velocities of 4 μm s^{-1} and 20 μm s^{-1}. We believe that this larger depletion is due to a stronger deformation of the STO surface during the tip's engagement and withdrawal from the surface. We have carefully excluded these regions while plotting ΔNVC in **b**. The scale bar in **a** represents 1 μm.

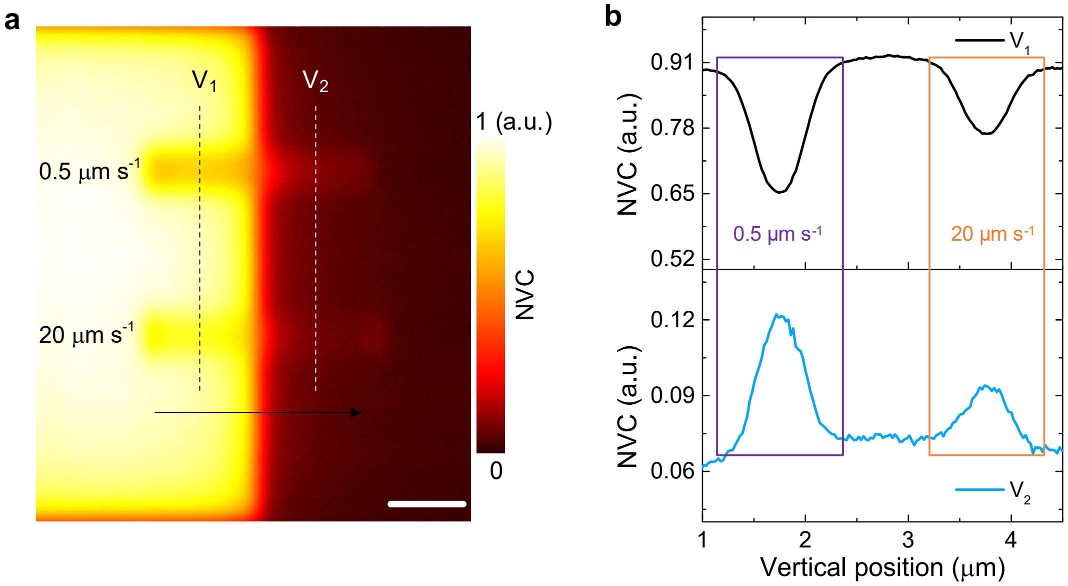

Supplementary Figure 11 | The scanning velocity dependence of vacancy redistribution. a, The normalised vacancy concentration (NVC) map after mechanical scans were performed with two different scanning velocities (0.5 and 20 µm s^{-1}) across the boundary between a $V_O^{\cdot\cdot}$-enriched and pristine regions. We used the 120-uc thick STO film and a sharp tip for this experiment. Scans were performed over a 3x0.5 μm^2 area with a contact force of 6 µN. The force was applied only during the trace, and the tip was lift-off during the retrace. Fives lines were traced from left-right as marked by the horizontal arrow. **b**, NVC profiles along lines V_1 and V_2 showing a decrease (increase) in vacancy concentration inside (outside) the vacancy enriched region. The profiles are averaged over a 0.5 µm wide window. For clarity, we marked the regions scanned with velocities 0.5 and 20 µm s^{-1} by purple and orange rectangles, respectively. Evidently, the net change in NVC inside and outside the vacancy enriched region is larger for the smaller scanning velocity. This implies the longer the tip stays in contact with the $V_O^{\cdot\cdot}$-enriched surface, a larger number of $V_O^{\cdot\cdot}$ migrate into the bulk from the contact point, and simultaneously an increased number of vacancies accumulate around the contact edge, yielding an enhanced lateral migration. Notably, unlike the experiments discussed in the main text (Figs. 2 and 4), where force was applied both during the race and retrace of the tip, in this controlled experiment the force was applied only during the trace. However, the NVC profiles in **b** clearly demonstrate the surface-bulk migration dominates over the lateral migration of vacancies with the tip. This observation further highlights the dominating role of the depolarisation field underneath a sharp tip. Note the region scanned with a tip velocity of 20 µm s^{-1} shows a large increase in NVC at the end of the trace. This is an artefact, caused by a larger deformation of the STO surface during the withdrawal of the tip from the surface. We often made similar observations even for an engagement of the tip on the pristine region. For profiling the NVC map in (**a**), we have thus excluded the left and right ends of the scanned regions. The scale bar in **a** represents 1 µm.

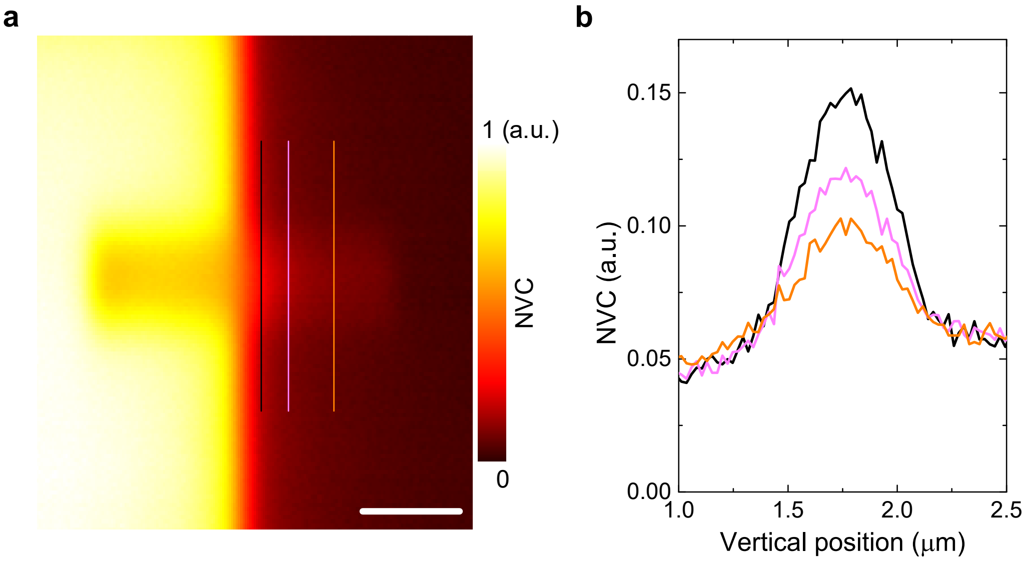

Supplementary Figure 12 | Evolution of vacancy concentration with distance from the vacancy-enriched region. a, Part of the normalised vacancy concentration (NVC) map shown in the supplementary figure 11. This image magnifies the region, which was scanned with a tip velocity of 0.5 μm s^{-1}. **b**, NVC profiles along lines marked on the NVC map in the black, red, and green colour, respectively. The lines are placed at a distance of about 0.2 μm, 0.5 μm, and 1 μm from the $V_o^{\cdot\cdot}$-enriched region. Evidently, the net gain in NVC systematically decreases with increasing distance from the $V_o^{\cdot\cdot}$-enriched region. We made a similar observation for the area scanned with the velocity of 20 μm s^{-1}. Note that the background NVC level in the pristine region uniformly decreases with increasing distance from the $V_o^{\cdot\cdot}$-enriched region. Thus, for this comparison we overlapped the background along the three lines in **a.** The scale bar in **a** represents 1 μm.

Supplementary Discussion: Role of the applied tip bias during KPFM imaging

Here, we address the influence of applied tip bias on KPFM images. We carried out this additional study to check whether the polarisation of STO film induced by the electric field from a biased tip affects the measured KPFM signal[18]. Note that for our work, we performed all KPFM measurements in the non-contact mode, whereby the tip and the STO surface were separated by an air-gap of 30 nm.

For an applied tip bias V (= V_{ac} + V_{dc}) and tip radius R, ignoring the radial distribution, the magnitude of the tip bias-induced electric field as a function of the tip-surface separation (lift height) z can be approximated as $E(z) = \frac{2RV}{z^2}$ [19]. Therefore, by comparing KPFM scans performed at different lift heights, we can readily assess how much the tip bias affects the measurement. Following this logic, we took KPFM images across a $V_o^{\cdot\cdot}$-enriched region of the 120-uc thick STO film by varying the lift height between 10-50 nm.

Supplementary Figs. 13 a-b show the representative KPFM image (taken at z = 10 nm) and corresponding KPFM profiles. Evidently, the KPFM signal of the pristine STO surface uniformly changes by about 100 mV upon decreasing z from 50 nm to 10 nm. This change is marginal, only 10% of the measured value (~ 1 V). Meanwhile, the KPFM contrast across the $V_o^{\cdot\cdot}$-enriched region shows a relatively larger but uniform change by about 500 mV, which is about 30% of the KPFM contrast obtained at z = 50 nm. Notably, during the KPFM imaging, the voltage (V_{dc}) that the feedback loop applies to the tip for nullifying the force between the tip and the STO surface corresponds to the measured KPFM signal. A simple algebraic calculation suggests that the strength of the tip bias-induced electric field should be 25 times larger at z = 10 nm than at 50 nm: E (z = 10 nm) = 25E (z = 50 nm). Comparatively, however, the corresponding change in the measured KPFM signal (within the pristine region) and KPFM contrast (across the $V_o^{\cdot\cdot}$-enriched region) is negligible.

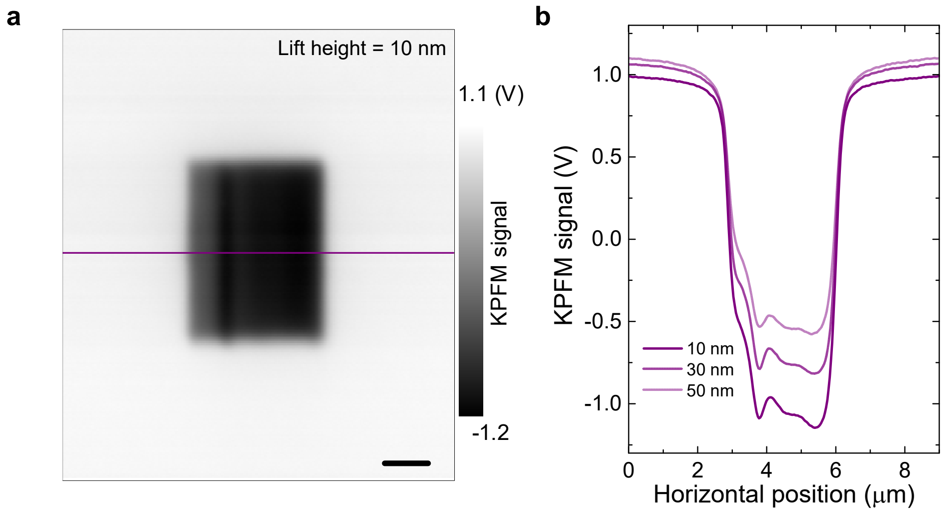

Supplementary Figure 13 | KPFM imaging by varying the lift height a, KPFM image of a $V_O^{\cdot\cdot}$-enriched STO surface. This image was obtained with a lift height of 10 nm. **b,** The KPFM profiles (along the line cut in **a**) from KPFM scans that were performed by varying the lift height between 10-50 nm. The scale bar in **a** represents 1 μm.

Before concluding this discussion, let us briefly comment on the relatively larger effect of *z*-variation on the KPFM contrast. $V_O^{\cdot\cdot}$ are electrically charged, and the electric field emanating from them would induce an electrostatic force between the tip and $V_O^{\cdot\cdot}$. The magnitude of this force would depend on *z*, and on the concentration of $V_O^{\cdot\cdot}$. The magnitude of this force, however, would be independent of the applied tip bias (*V*) [20]. Thus, during KPFM imaging, the closer the tip approaches towards the $V_O^{\cdot\cdot}$-enriched region, the stronger it feels this $V_O^{\cdot\cdot}$-induced force. Accordingly, the feedback loop utilizes a larger V_{dc} for nullifying the force acting between the tip and the $V_O^{\cdot\cdot}$-enriched STO surface. These considerations readily corroborate the relatively larger effect of *z*-variation on the KPFM contrast.

To sum up, based on our experiments we conclude that the influence of the applied tip bias and the resulting polarisation of STO is rather weak and, more importantly, is uniform across an entire KPFM image. While defining the concentration of $V_O^{\cdot\cdot}$ or creating NVC maps from KPFM images, we defined the concentration of $V_O^{\cdot\cdot}$ to be equal to the difference between the KPFM signals measured from the pristine and $V_O^{\cdot\cdot}$-enriched regions. This approach readily removes this uniform contribution of the tip bias.

Supplementary References

1. Schie, M., Marchewka, A., Müller, T., De Souza, R. a & Waser, R. Molecular dynamics simulations of oxygen vacancy diffusion in $SrTiO_3$. *J. Phys. Condens. Matter* **24,** 485002 (2012).

2. Andrä, M. *et al.* The influence of the local oxygen vacancy concentration on the piezoresponse of strontium titanate thin films. *Nanoscale* **7,** 14351–14357 (2015).

3. Bieger, T., Maier, J. & Waser, R. Kinetics of oxygen incorporation in $SrTiO_3$ (Fe-doped): an optical investigation. *Sensors Actuators B Chem.* **7,** 763–768 (1992).

4. Merkle, R. & Maier, J. How is oxygen incorporated into oxides? A comprehensive kinetic study of a simple solid-state reaction with $SrTiO_3$ as a model material. *Angew. Chemie Int. Ed.* **47,** 3874–3894 (2008).

5. Lee, K. Y. *et al.* Controllable charge transfer by ferroelectric polarization mediated triboelectricity. *Adv. Funct. Mater.* **26,** 3067–3073 (2016).

6. Müller, K. A. & Burkard, H. $SrTiO_3$: An intrinsic quantum paraelectric below 4 K. *Phys. Rev. B* **19,** 3593–3602 (1979).

7. Haeni, J. H. *et al.* Room-temperature ferroelectricity in strained $SrTiO_3$. *Nature* **430,** 758–761 (2004).

8. Kim, Y. S. *et al.* Localized electronic states induced by defects and possible origin of ferroelectricity in strontium titanate thin films. *Appl. Phys. Lett.* **94,** 202906 (2009).

9. Lee, D. *et al.* Emergence of room-temperature ferroelectricity at reduced dimensions. *Science* **349,** 1314–1317 (2015).

10. Lu, H. *et al.* Mechanical writing of ferroelectric polarization. *Science* **336,** 59–61 (2012).

11. Li, Y. L. *et al.* Phase transitions and domain structures in strained pseudocubic (100) $SrTiO_3$ thin films. *Phys. Rev. B* **73,** 184112 (2006).

12. Jesse, S., Baddorf, A. P. & Kalinin, S. V. Dynamic behaviour in piezoresponse force microscopy. *Nanotechnology* **17,** 1615–1628 (2006).

13. Balke, N. *et al.* Exploring local electrostatic effects with scanning probe microscopy: Implications for piezoresponse force microscopy and triboelectricity. *ACS Nano* **8,** 10229–10236 (2014).

14. Stengel, M. Surface control of flexoelectricity. *Phys. Rev. B* **90,** 201112 (2014).

15. Narvaez, J., Saremi, S., Hong, J., Stengel, M. & Catalan, G. Large flexoelectric anisotropy in paraelectric barium titanate. *Phys. Rev. Lett.* **115,** 37601 (2015).

16. Alexandrov, V. M. & Pozharskii, D. A. *Three-dimensional contact problems*. (Springer Netherlands, 2001).

17. Moos, R. & Hardtl, K. H. Defect chemistry of donor-doped and undoped strontium titanate ceramics between 1000° and 1400°C. *J. Am. Ceram. Soc.* **80,** 2549–2562 (1997).

18. Nielsen, D. A., Popok, V. N. & Pedersen, K. Modelling and experimental verification of tip-induced polarization in Kelvin probe force microscopy measurements on dielectric surfaces. *J. Appl. Phys.* **118,** 195301 (2015).

19. Gruverman, A., Auciello, O. & Tokumoto, H. Imaging and control of domain structures in ferroelectric thin films via scanning force microscopy. *Annu. Rev. Mater. Sci.* **28,** 101–123 (1998).

20. Orihuela, M. F., Somoza, A. M., Colchero, J., Ortuño, M. & Palacios-Lidón, E. Localized charge imaging with scanning Kelvin probe microscopy. *Nanotechnology* **28,** 25703 (2017).
